# Post-cooling fever in post-cardiac arrest patients: post-cooling normothermia as part of target temperature management?

**DOI:** 10.1186/1471-227X-15-S1-A8

**Published:** 2015-06-24

**Authors:** Cathy De Deyne

**Affiliations:** 1 Department of Anesthesiology and Critical Care Medicine, Ziekenhuis Oost-Limburg Genk, Belgium; Faculty of Medical Life Sciences, University Hasselt UH, Hasselt, Belgium

## 

Experimental studies support that fever after ischaemic brain injury may not only be a surrogate marker for severe cerebral ischaemia but may also deteriorate pre-existent cerebral ischaemic damage and should therefore be treated [1,2]. Neurological damage after cardiac arrest (CA) still determines the final outcome and post-CA critical care focuses on maximal neuroprotection, the application of therapeutic hypothermia (TH). Since the use of TH, many reports have been published on the occurrence of fever post TH or so-called post-cooling fever (PCF). However, the incidence of post-CA fever without TH is similar, ranging from 20 to 78%, suggesting that episodes of temperature >38°C are likely to occur in all patients after CA irrespective of whether TH was administered.

Bro-Jeppesen and colleagues reported a higher 30-day mortality in patients with PCF (temperature >38.5°C) compared with patients without (36% vs. 22%) [3]. Likewise, 1-year unfavourable neurological outcome (43% vs. 27%) was higher in patients with PCF compared with patients without. Maximum temperature and PCF duration were independent predictors of mortality. Their PCF incidence (50.4%, 136/270) was high, probably explained by a longer observation period (36 hours). Leary and colleagues reported a PCF incidence of 41% (69/176) within 24 hours after rewarming [4], while Gebhardt and colleagues reported a 42% incidence (141/336) within 48 hours after CA [5] (both defined PCF as temperature >38°C).

Most importantly, recent data suggest that the effect on mortality becomes significant only with PCF >39°C and a minimum duration of 7 hours. Similarly, Leary and colleagues did not find any effect of PCF on survival and neurological outcome, but a maximum temperature >38.7°C was associated with worse outcome. A prospective analysis of our own post-CA data confirmed this increased mortality in the presence of PCF only above 39°C. In our population of 76 out-of-hospital post-CA patients, PCF between 38 and 39°C did not influence outcome.

The question remains whether we should actively treat (or even prevent) PCF. Many currently applied TH protocols do include a post-cooling period (until 36 hours after rewarming) of induced normothermia (37°C by endovascular or surface cooling) [6]. But is this prolonged normothermia practice improving the (neurological) outcome of our patient? There are arguments in favour of a correlation between high PCF (above 39°C) and post-CA outcome. However, whether this high fever is only an epiphenomenon of the severity of cerebral ischaemic injury and whether outcome can be improved by application of strict normothermia in the early post-cooling hours is still undetermined.

## Financial disclosure

CDD has received speaker’s reimbursements from C. R. BARD.

## Declaration

This abstract and supplement was proposed, developed and commissioned by BMC Emergency Medicine and was funded by an educational grant from C. R. BARD, NJ, USA. The published abstract was independently prepared by the author. C. R. BARD had no input into the content.
